# Comparative efficacy and safety of suvorexant and lemborexant for insomnia treatment

**DOI:** 10.1002/pcn5.85

**Published:** 2023-03-21

**Authors:** Teruaki Hayashi, Takehiko Yamanashi, Masaaki Iwata

**Affiliations:** ^1^ Department of Neuropsychiatry Faculty of Medicine Tottori University Yonago Japan

**Keywords:** efficacy, lemborexant, orexin receptor antagonist, side‐effects, suvorexant

## Abstract

**Aim:**

Although suvorexant and lemborexant, which have orexin receptor antagonist activity, are used as sleep medications in Japan, no report has directly compared their efficacy and safety. This study compared the efficacy and safety of the drugs.

**Methods:**

This retrospective cohort study included patients who presented to the Outpatient Department of Psychiatry at Tottori University Hospital between December 1, 2020, and December 31, 2021. Information was obtained from 108 patients who were newly treated with suvorexant or lemborexant. Data were analyzed after excluding one case of discontinuation due to a post‐administration allergic reaction. Improvement in sleep status after administration was assessed retrospectively from medical records by using the Clinical Global Impressions‐Improvement (CGI‐I) Scale, which is a subscale of the Clinical Global Impressions (CGI) Scale. The incidence of side‐effects was obtained from the medical records of the patient's first visit after administration.

**Results:**

There was no significant difference between the CGI‐I scores in the suvorexant (mean [SD], 3.05 [0.93]) and lemborexant groups (mean [SD], 3.38 [0.83]) (*p* = 0.10). The incidence of side‐effects with continued treatment was not significantly different between the suvorexant group (12.5%) and the lemborexant group (2.9%) (*p* = 0.10). Patients who switched from suvorexant to lemborexant had CGI‐I scores ≤4, and no side‐effects were observed after switching to lemborexant.

**Conclusion:**

There was no difference in effectiveness between suvorexant and lemborexant. However, lemborexant might cause side‐effects less frequently than suvorexant, at least in the early stages of treatment.

## INTRODUCTION

In the pharmacotherapy of insomnia using GABA receptor agonists, adverse events associated with long‐term use, such as falls, cognitive impairment, abuse, and dependence, have become a concern. Dual orexin receptor antagonists (DORAs), which have a mechanism of action that is not mediated by GABA receptors, were developed to address this issue. One study indicated that cognitive improvement was achieved after switching from benzodiazepine to DORAs.^1^

In Japan, suvorexant and lemborexant were launched in 2014 and July 2020, respectively. Among the orexin receptors, orexin 2 receptors are related to sleep.^2^, ^3^ Suvorexant acts more strongly on orexin 1 receptors, whereas lemborexant acts more strongly on orexin 2 receptors.

Considering that suvorexant has been in the market for several years, there have been many reports on its efficacy and safety.^3^, ^4^ However, because lemborexant has only been in the market for 2 years, post‐marketing surveillance data is insufficient. Furthermore, as far as we have been able to ascertain, no study has been published that directly compares the efficacy and safety of these two drugs,^5^ but meta‐analyses exist on this topic.^6^

In this study, we conducted a retrospective comparison of the efficacy and safety of suvorexant and lemborexant in the Outpatient Department of Psychiatry at Tottori University Hospital by using medical records.

## METHODS

### Study design

This is a single‐center retrospective observational study. Information was collected from electronic medical records. The opportunity for the research participant to refuse (opt out) was guaranteed instead of consent. This study was conducted in compliance with the ethical guidelines for medical research involving humans and was approved by the Faculty of Medicine Tottori University Ethical Review Committee (approval number: 22A052).

### Patients

This study included patients who attended the Outpatient Department of Psychiatry at Tottori University Hospital between December 1, 2020, and December 31, 2021. From these patients, those newly treated with suvorexant or lemborexant were selected. Patients who switched from one DORA to another were excluded from the analysis comparing suvorexant and lemborexant and were described separately.

### Survey and assessment items

#### Patient characteristics

Data on patient characteristics, including age, sex, medication, and psychiatric comorbidities, were collected from the medical records of the outpatient department. Diazepam (DAP), chlorpromazine (CP), and imipramine (I) equivalents were calculated if anxiolytics, antipsychotic drugs, or antidepressants were used prior to the introduction of suvorexant and lemborexant. The equivalent conversion table developed by Inagaki and Inada was used to calculate each conversion dose.^6^

#### Efficacy and safety

Sleep status before DORA administration was assessed using the Clinical Global Impressions‐Severity (CGI‐S) Scale, and the degree of improvement after DORA administration was assessed using the Clinical Global Impressions‐Improvement (CGI‐I) Scale; the CGI‐S and CGI‐I are subscales of the Clinical Global Impressions (CGI) Scale.^7^ CGI‐S and CGI‐I were evaluated retrospectively as previously reported^8^, ^9^ by the author (T. H.), who is a specialist in the Japanese Society of Psychiatry and Neurology. The CGI‐S was scored on the basis of the patients’ medical records at the outpatient clinic visit immediately prior to DORA administration. The CGI‐I was scored by comparing sleep status at the visit immediately prior to DORA administration with the sleep status at the very first visit after DORA administration. Information on the incidence of side‐effects was collected from the medical records at the patient's very first visit after DORA administration. The CGI‐I and side‐effects were evaluated at the same time by using the patient's medical records at the first visit after administration.

### Statistics analyses

Fisher's exact test was used for sex comparisons. The *t*‐test was used for age and DAP, CP, and I equivalents. The Mann–Whitney *U* test was used for the CGI‐S scores. For each endpoint, the Mann–Whitney *U* test was used for CGI‐I scores and Fisher's exact test was used to compare the incidence of adverse events. Furthermore, patients diagnosed with F2 (schizophrenia, schizotypal, and delusional disorders), F3 (mood disorders), and F4 (neurotic, stress‐related, and somatoform disorders) per the *International Statistical Classification of Diseases and Related Health Problems* 10th Revision^10^ were evaluated using the CGI‐I and side‐effects separately.

All statistical analyses were performed with EZR Version 1.52 (Saitama Medical Center, Jichi Medical University), a graphical user interface for R Version 4.02 (The R Foundation for Statistical Computing). More precisely, it is a modified version of R‐Commander designed to add statistical functions frequently used in biostatistics.^11^

## RESULTS

### Participant demographics

A total of 108 patients who were newly administered with DORA were included. Forty patients were newly prescribed suvorexant (suvorexant group), and 68 patients were newly prescribed lemborexant (lemborexant group). Thirteen patients were switched from suvorexant to lemborexant. In one case, DORA was discontinued because of an allergic reaction after DORA administration. No patient had previously received lemborexant at the time of suvorexant prescription.

The male/female ratio was 12/28 and 33/35 in the suvorexant and lemborexant groups, respectively. The mean age was 42.0 (SD = 17.9) and 46.2 (SD = 16.7) years in the suvorexant and lemborexant groups, respectively. Most eligible cases were F2, F3, and F4 in both groups. No case in the suvorexant, and seven cases in the lemborexant groups were categorized as F5 (behavioral syndromes associated with physiological disturbances and physical factors), but they were diagnosed with insomnia without any other psychiatric disorders.

The mean CGI‐S scores were 3.85 (SD = 0.36) and 3.92 (SD = 0.36) in the suvorexant and lemborexant groups, respectively, with no significant difference (*p* = 0.30). Pre‐administration psychotropic drug use in each group was calculated and expressed as DAP, CP, and I equivalents. The mean starting dose for each group was 4.6 (SD = 7.7) and 6.9 (SD = 8.4) mg/day for the DAP equivalent in the suvorexant group and lemborexant groups, respectively (*p* = 0.15). The CP equivalents were 94.5 (SD = 198.3) and 147.0 (SD = 236.1) mg/day in the suvorexant and lemborexant groups, respectively (*p* = 0.24). The I equivalents were 62.5 (SD = 114.3) and 51.8 (SD = 104.8) mg/day in the suvorexant and lemborexant groups, respectively (*p* = 0.62).

### Efficacy

There was no significant difference between CGI‐I in the suvorexant group (mean 3.05 [SD 0.93]) and CGI‐I in the lemborexant group (mean 3.38 [SD 0.83]) (*p* = 0.10) (Table 1). Thereafter, we performed subgroup analysis for patients diagnosed as F2, F3, or F4. Subgroup analyses were performed to account for the possibility that the therapeutic effects or side‐effects of DORA differ by patient disease/disorder. The subgroup analyses were limited to subjects in these three categories because the number of patients with other diagnoses was very limited. No significant difference between CGI‐I scores in the suvorexant and lemborexant groups was observed in F2 or F3 patients (Tables 2 and 3). In F4 patients, the CGI‐I score was higher in the lemborexant group (mean 3.47 [SD 0.61]) than in the suvorexant group (mean 2.85 [SD 0.80]) (*p* = 0.03, no correction) (Table 4).

**Table 1:** Patient characteristics.

Classification	All subjects	Drug			
		Suvorexant	Lemborexant		
*N*	108	40	68	*p*	Statistical test
Mean age (years)	44.6	42.0	46.2	0.22	*t* = 1.23
SD	17.2	17.9	16.7		
Female sex (*n*)	63	28	35	0.07	Fisher's exact
%	56.8	70.0	51.5		
CGI‐S score *n* (%)				0.30	Mann–Whitney *U*
1	0 (0.0)	0 (0.0)	0 (0.0)		*W* = 1458
2	0 (0.0)	0 (0.0)	0 (0.0)		
3	13 (12.0)	6 (15.0)	7 (10.3)		
4	93 (86.1)	34 (85.0)	59 (86.8)		
5	2 (1.9)	0 (0.0)	2 (2.9)		
Mean CGI‐S	3.9	3.85	3.92		
SD	0.36	0.36	0.36		
Dose mg/*n*		10 mg/3	2.5 mg/8		
		15 mg/16	5 mg/50		
		20 mg/15	10 mg/1		
		p.r.n.15 mg/1	p.r.n.2.5 mg/8		
		p.r.n.20 mg/5	p.r.n.5 mg/1		
Mental disorder *n* (%)					
F0	4 (3.3)	0 (0.0)	4 (5.9)		
F1	1 (0.8)	1 (2.5)	0 (0.0)		
F2	19 (17.1)	7 (17.5)	12 (17.6)		
F3	31 (27.9)	11 (27.5)	20 (29.4)		
F4	32 (28.8)	13 (32.5)	19 (27.9)		
F5	7 (6.3)	0 (0.0)	7 (10.3)		
F6	0 (0.0)	0 (0.0)	0 (0.0)		
F7	2 (1.8)	2 (5.0)	0 (0.0)		
F8	2 (1.8)	1 (2.5)	1 (1.5)		
F9	7 (6.3)	2 (5.0)	5 (7.4)		
*G*	3 (2.7)	3 (7.5)	0 (0.0)		
Mean diazepam equivalent	6.1	4.6	6.9	0.15	*t* = 1.44
SD	8.2	7.7	8.4		
Mean chlorpromazine equivalent	127.5	94.5	147.0	0.24	*t* = 1.18
SD	223.4	198.3	236.1		
Mean imipramine equivalent	55.8	62.5	51.8	0.62	*t* = −0.49
SD	108.0	114.3	104.8		
CGI‐I score *n* (%)				0.10	Mann–Whitney *U*
1	2 (1.8)	2 (5.0)	0 (0.0)		*W* = 1606
2	22 (19.6)	10 (25.0)	12 (17.6)		
3	33 (29.5)	12 (30.0)	21 (30.9)		
4	48 (42.9)	16 (40.0)	32 (47.1)		
5	3 (2.7)	0 (0.0)	3 (4.4)		
Mean CGI‐I	3.26	3.05	3.38		
SD	0.88	0.93	0.83		
Safety					
Side‐effects (*n*)	7	5	2	0.10	Fisher's exact
%	6.5	12.5	2.9		
Side‐effects—headache (*n*)	4	2	2	0.63	Fisher's exact
%	3.7	5.0	2.9		

Abbreviations: CGI‐I, Clinical Global Impressions‐Improvement; CGI‐S, Clinical Global Impressions‐Severe; p.r.n. (pro re nata), according to need.

**Table 2:** Efficacy and safety of suvorexant and lemborexant in F2.

Classification	All subjects	Drug			
		Suvorexant	Lemborexant		
*N*	19	7	12	*p*	Statistical test
Mean age (years)	43.6	42.1	44.4	0.70	*t* = 0.39
SD	12.1	12.3	12.3		
Female sex (*n*)	12	6	6	0.17	Fisher's exact
%	63.2	85.7	50.0		
CGI‐S score *n* (%)				0.29	Mann–Whitney *U*
1	0 (0.0)	0 (0.0)	0 (0.0)		*W* = 50.5
2	0 (0.0)	0 (0.0)	0 (0.0)		
3	3 (15.8)	2 (28.6)	1 (8.3)		
4	16 (84.2)	5 (71.4)	11 (91.7)		
5	0 (0.0)	0 (0.0)	0 (0.0)		
Mean CGI‐S	3.84	3.71	3.92		
SD	0.37	0.49	0.29		
Mean diazepam equivalent	7.8	5.0	9.5	0.35	*t* = 0.96
SD	9.7	5.8	11.3		
Mean chlorpromazine equivalent	372.5	442.9	331.5	0.45	*t* = −0.77
SD	301.0	257.3	327.4		
Mean imipramine equivalent	71.1	85.7	62.5	0.81	*t* = −0.25
SD	189.5	226.8	174.7		
CGI‐I score *n* (%)				0.09	Mann–Whitney *U*
1	1 (5.3)	1 (14.3)	0 (0.0)		*W* = 61
2	6 (31.6)	3 (42.9)	3 (25.0)		
3	2 (10.5)	1 (14.3)	1 (8.3)		
4	9 (47.4)	2 (28.6)	7 (58.3)		
5	1 (5.3)	0 (0.0)	1 (8.3)		
Mean CGI‐I	3.16	2.57	3.50		
SD	1.09	1.14	1.00		
Safety					
Side‐effects (*n*)	0	0	0		
%	0.0	0.0	0.0		
Side‐effects—headache (*n*)	0	0	0		
%	0.0	0.0	0.0		

Abbreviations: CGI‐I, Clinical Global Impressions‐Improvement; CGI‐S, Clinical Global Impressions‐Severe.

**Table 3:** Efficacy and safety of suvorexant and lemborexant in F3.

Classification	All subjects	Drug			
		Suvorexant	Lemborexant		
*N*	31	11	20	*p*	Statistical test
Mean age (years)	50.6	53.3	49.2	0.57	*t* = −0.58
SD	18.5	21.0	17.4		
Female sex (*n*)	19	9	10	0.13	Fisher's exact
%	61.3	81.8	50.0		
CGI‐S score *n* (%)				0.26	Mann–Whitney *U*
1	0 (0.0)	0 (0.0)	0 (0.0)		*W* = 124.5
2	0 (0.0)	0 (0.0)	0 (0.0)		
3	3 (9.7)	2 (18.2)	1 (5.0)		
4	28 (90.3)	9 (81.8)	19 (95.0)		
5	0 (0.0)	0 (0.0)	0 (0.0)		
Mean CGI‐S	3.90	3.81	3.95		
SD	0.30	0.40	0.22		
Mean diazepam equivalent	6.4	4.0	7.8	0.21	*t* = 1.28
SD	8.2	4.8	9.4		
Mean chlorpromazine equivalent	135.7	16.0	201.6	0.03*	*t* = 2.25
SD	234.0	31.7	270.3		
Mean imipramine equivalent	53.0	73.3	41.9	0.20	*t* = −1.32
SD	64.4	60.2	65.3		
CGI‐I score *n* (%)				0.74	Mann–Whitney *U*
1	0 (0.0)	0 (0.0)	0 (0.0)		*W* = 118
2	5 (016.1)	2 (18.2)	3 (15.0)		
3	9 (29.0)	3 (27.3)	6 (30.0)		
4	15 (48.4)	6 (54.5)	9 (45.0)		
5	2 (6.5)	0 (0.0)	2 (10.0)		
Mean CGI‐I	3.45	3.36	3.50		
SD	0.85	0.81	0.89		
Safety					
Side‐effects (*n*)	4	3	1	0.12	Fisher's exact
%	12.9	27.3	5.0		
Side‐effects—headache (*n*)	3	2	1	0.28	Fisher's exact
%	9.7	18.2	5.0		

Abbreviations: CGI‐I, Clinical Global Impressions‐Improvement; CGI‐S, Clinical Global Impressions‐Severe.

* *p* < 0.05.

**Table 4:** Efficacy and safety of suvorexant and lemborexant in F4.

Classification	All subjects	Drug			
		Suvorexant	Lemborexant		
*N*	32	13	19	*p*	Statistical test
Mean age (years)	44.6	39.2	48.2	0.14	*t* = 1.54
SD	16.6	16.5	16.1		
Female sex (*n*)	16	7	9	1	Fisher's exact
%	50.0	53.8	47.4		
CGI‐S score *n* (%)				0.80	Mann–Whitney *U*
1	0 (0.0)	0 (0.0)	0 (0.0)		*W* = 128.5
2	0 (0.0)	0 (0.0)	0 (0.0)		
3	5 (15.6)	2 (15.4)	3 (15.8)		
4	26 (81.3)	11 (84.6)	15 (78.9)		
5	1 (3.1)	0 (0.0)	1 (5.3)		
Mean CGI‐S	3.88	3.85	3.89		
SD	0.42	0.38	0.46		
Mean diazepam equivalent	5.7	5.4	5.8	0.87	*t* = 0.165
SD	7.5	10.1	5.5		
Mean chlorpromazine equivalent	49.8	38.7	57.4	0.62	*t* = 0.51
SD	101.7	85.9	112.8		
Mean imipramine equivalent	72.9	84.1	65.1	0.60	*t* = −0.52
SD	99.6	99.3	101.8		
CGI‐I score *n* (%)				0.03*	Mann–Whitney *U*
1	0 (0.0)	0 (0.0)	0 (0.0)		*W* = 177.5
2	6 (18.8)	5 (38.5)	1 (5.3)		
3	13 (40.6)	5 (38.5)	8 (42.1)		
4	13 (40.6)	3 (23.1)	10 (52.6)		
5	0 (0.0)	0 (0.0)	0 (5.0)		
Mean CGI‐I	3.22	2.85	3.47		
SD	0.75	0.80	0.61		
Safety					
Side‐effects (*n*)	2	1	1	1	Fisher's exact
%	6.3	7.7	5.3		
Side‐effects—headache (*n*)	1	0	1	1	Fisher's exact
%	3.1	0.0	5.3		

Abbreviations: CGI‐I, Clinical Global Impressions‐Improvement; CGI‐S, Clinical Global Impressions‐Severe.

* *p* < 0.05.

### Safety

In one case, the administration of lemborexant was discontinued because of an allergic reaction. Side‐effects were examined in 108 patients who were able to continue their medication. There were no cases in which the drug was continued despite allergic reactions.

The incidence of side‐effects was higher in the suvorexant group (5 of 40 patients [12.5%]) than in the lemborexant group (2 of 68 patients [2.9%]), but without statistical significance (Fisher's exact test, *p* = 0.10). In the suvorexant group, headache occurred in two patients (5.0%), carry‐over effects to the next day in two patients (5.0%), and nightmares in one patient (2.5%). In the lemborexant group, headache occurred in two patients (2.9%).

### Cases changed from a DORA to another type of DORA

We found 13 patients who switched from suvorexant to lemborexant. In those 13 patients, the mean CGI‐I was 2.77 ± 0.93. The CGI‐I scores of all patients were ≤4; therefore, no patient was evaluated as “worse” after switching to lemborexant. No side‐effects were observed after switching to lemborexant (Table 5). No patient switched from lemborexant to suvorexant.

**Table 5:** Efficacy and safety in 13 cases of switching from suvorexant to lemborexant.

Classification	All subjects													
*N*	13	1	2	3	4	5	6	7	8	9	10	11	12	13
Age (years)	44.6 ± 17.2	45	43	30	41	32	65	31	49	24	46	74	33	39
Female sex (%)	7 (53.8)	Male	Male	Female	Female	Female	Female	Male	Female	Male	Male	Female	Female	Male
CGI‐S score	4.08 ± 0.28	4	4	4	4	4	4	4	4	4	4	5	4	4
Dose (mg)	4.8 ± 0.7	5	5	5	5	5	5	5	2.5	5	5	5	5	5
Mental disorder		2	7	9	7	2	4	8	3	5	7	3	2	7
Mean diazepam equivalent	6.1 ± 8.2	28.5	45	9.3	0	0	10	4	6	15	85	0	0	10
Mean chlorpromazine equivalent	127.5 ± 223.4	550.0	1061	0	75	200	0	25	37.5	0	0	237.9	300	50
Mean imipramine equivalent	55.8 ± 108.0	0.0	0	200	0	0	0	0	93.8	75	0	0	0	0
CGI‐I score	2.77 ± 0.93	4	2	2	3	2	4	3	2	2	2	4	4	2
Safety		None	None	None	None	None	None	None	None	None	None	None	None	None

Abbreviations: CGI‐I, Clinical Global Impressions‐Improvement; CGI‐S, Clinical Global Impressions‐Severe.

## DISCUSSION

Orexin receptors include orexin 1 and 2 receptors,^4^ and suvorexant was launched as a drug that induces sleep by inhibiting both orexin receptor types. Subsequently, it was found that the orexin 2 receptor is more involved in sleep. In anticipation of this finding, lemborexant was launched.^12^ Therefore, we hypothesized that there is a difference in efficacy or side‐effects between the two drugs.

First, we investigated the medication efficacy using the CGI‐I score, which showed no significant difference in all participants. However, when we examined the results for each mental disorder, we found that the CGI‐I scores of the F4 patients were lower in the lemborexant group than in the suvorexant groups with a small *p*‐value (*p* = 0.03). It is impossible to say definitively because *p*‐values were not corrected for this subgroup analyses, but lemborexant might improve a patient's sleep status more than suvorexant in F4 patients. Patient backgrounds were compared to examine what influenced the difference in efficacy, which showed no significant differences (Table 4). Furthermore, among the 13 patients who switched from suvorexant to lemborexant, all patients were assessed as “neither improved nor worse” or “improved.” In other words, no patient was assessed as “worse” after switching from suvorexant to lemborexant. Next, we compared the differences in side‐effects between the suvorexant and lemborexant groups. Although there was not statistical significance, overall side‐effects, including headache, carry‐over, and nightmare, were more frequently observed in the suvorexant group. Furthermore, no side‐effects were observed after switching from suvorexant to lemborexant in the 13 patients. These results suggest the possibility that lemborexant might cause side‐effects less frequently than suvorexant, at least in the early stages of treatment.

Several studies have investigated the clinical features of lemborexant use. An improvement in difficulty initiating sleep was observed after switching from suvorexant to lemborexant.^13^ Lemborexant is well tolerated and is in Schedule IV under the US Drug Enforcement Administration's Controlled Substances Act; as suvorexant had been placed. Compared with Schedule III drugs, including, barbiturates, phencyclidine, and ketamine, Schedule IV drugs have lower abuse potential and are considered less dangerous.^14^ Regarding cognitive function, lemborexant and suvorexant do not adversely affect cognitive function in patients with mild to moderate Alzheimer's disease and sleep disturbances; as assessed by the Mini Mental State Examination, neither lemborexant nor suvorexant adversely affected cognitive function.^15^, ^16^ Several network meta‐analyses compared the efficacy and safety of lemborexant and suvorexant.^17^, ^18^, ^19^ One study found no significant differences in efficacy or safety between the two drugs.^17^ However, Kishi et al. reported that 10 mg of lemborexant was superior to 20 or 15 mg of suvorexant in increasing subjective total sleep time and reducing subjective wake time after sleep onset (sWASO) and subjective time to sleep onset (sTSO), but that it was inferior with a higher discontinuation due to adverse events.^18^ Xue et al. also showed that the effect sizes of lemborexant for sWASO and sTSO were larger than those of suvorexant in their network meta‐analysis.^19^ However, no previous reports have directly compared the efficacy and side‐effects between lemborexant and suvorexant. Further study will be needed to clarify which drug can be used with better effects and fewer side‐effects.

### Limitations

This study has several limitations. First, only one evaluator scored the CGIs. CGI‐S and CGI‐I scorings are easily influenced by evaluator subjectivity and are not reproducible because they have only a simple single‐item structure. Second, we used the CGI‐I scores instead of a sleep‐specific index to evaluate the efficacy of each drug. Generally, the CGI score has been used to evaluate global function of patients with psychiatric disorders.^7^ Recently, several reports showed that the CGI score was a potential substitute for conventional rating scales among patients with depression or schizophrenia.^20^, ^21^ Furthermore, it has recently been expanded to cover a variety of disorders and symptoms, including sleep status.^22^, ^23^ However, in the future, this should be performed using the Athens Insomnia Scale,^23^ which is commonly used in research settings. The third limitation is that the scores were not assessed at the pre‐ and post‐dose outpatient visits; rather, the information was collected retrospectively from medical records. Further prospective study using sleep‐specific rating scales will be needed to validate the difference between the effects of suvorexant and lemborexant. Fourth, the CGI‐I and side‐effects were evaluated simultaneously from the medical records at the first post‐administration visit. Almost all cases in the study had their first post‐administration visit set 2–4 weeks after administration, which is a short evaluation period, and the duration varies by patient. Furthermore, long‐term efficacy and safety were not evaluated. Fifth, the sample size was small. This was because lemborexant has only been in the market for 1 year. Finally, all participants were Asian because this survey was conducted at a single institution. Studies with more participants and multicenter studies are needed to generalize these results.

## AUTHOR CONTRIBUTIONS

Teruaki Hayashi collected and organized the clinical data, analyzed the initial preparation, and wrote the initial draft of the manuscript. Takehiko Yamanashi analyzed the data and critically reviewed the manuscript. Masaaki Iwata conceived the study, planned its design and coordination, and edited the final form of the manuscript.

## CONFLICT OF INTEREST STATEMENT

The authors declare that there are no conflicts of interest related to this study. We have had the following interests over the past 3 years: Takehiko Yamanashi has received grant funding from the Takeda Science Foundation and Mochida Memorial Foundation and speaker's honoraria from Viatris, Sumitomo Pharma, and MSD K.K.; Masaaki Iwata has received grant funding from the Japan Society for the Promotion of Science, SENSHIN Medical Research Foundation, Japan Agency for Medical Research and Development, Osaka Gas, and speaker's honoraria from Viatris, Sumitomo Pharma, Otsuka, Meiji‐Seika Pharma, Eli Lilly, MSD K.K., Eisai, Pfizer, Janssen Pharmaceutical, Mochida Pharmaceutical, Takeda Pharmaceutical, and Yoshitomiyakuhin.

## ETHICAL APPROVAL STATEMENT

This study was conducted in compliance with the ethical guidelines for medical research involving humans and was approved by the Faculty of Medicine Tottori University Ethical Review Committee (approval number: 22A052).

## PATIENT CONSENT STATEMENT

The opportunity of the research participant to refuse (opt out) was guaranteed instead of obtaining consent.

## CLINICAL TRIAL REGISTRATION

N/A.

## Data Availability

The data supporting the findings of this study are available from the corresponding author, Takehiko Yamanashi, upon reasonable request.
